# B2M is a Biomarker Associated With Immune Infiltration In High Altitude Pulmonary Edema

**DOI:** 10.2174/1386207326666230510095840

**Published:** 2023-05-15

**Authors:** Mu Yuan, Xueting Hu, Wei Xing, Xiaofeng Wu, Chengxiu Pu, Wei Guo, Xiyan Zhu, Mengwei Yao, Luoquan Ao, Zhan Li, Xiang Xu

**Affiliations:** 1 Department of Stem Cell and Regenerative Medicine, State Key Laboratory of Trauma, Burn and Combined Injury, Daping Hospital, Army Medical University, 400010, Chongqing, China;; 2 Central Laboratory, State Key Laboratory of Trauma, Burn and Combined Injury, Daping Hospital, Army Medical University, 400010, Chongqing, China;; 3 Department of Military Traffic Injury Prevention and Treatment, Daping Hospital, Army Medical University, 400010, Chongqing, China

**Keywords:** High altitude pulmonary edema, early diagnosis, pathogenesis, biomarkers, hub genes, B2M, immune infiltration, WGCNA

## Abstract

**Background:**

High altitude pulmonary edema (HAPE) is a serious mountain sickness with certain mortality. Its early diagnosis is very important. However, the mechanism of its onset and progression is still controversial.

**Aim:**

This study aimed to analyze the HAPE occurrence and development mechanism and search for prospective biomarkers in peripheral blood.

**Methods:**

The difference genes (DEGs) of the Control group and the HAPE group were enriched by gene ontology (GO), Kyoto Encyclopedia of Genes and Genomes (KEGG) enrichment analysis, and then GSEA analysis was performed. After identifying the immune-related hub genes, QPCR was used to verify and analyze the hub gene function and diagnostic value with single-gene GSEA and ROC curves, and the drugs that acted on the hub gene was found in the CTD database. Immune infiltration and its association with the hub genes were analyzed using CIBERSORT. Finally, WGCNA was employed to investigate immune invasion cells' significantly related gene modules, following enrichment analysis of their GO and KEGG.

**Results:**

The dataset enrichment analysis, immune invasion analysis and WGCNA analysis showed that the occurrence and early progression of HAPE were unrelated to inflammation. The hub genes associated with immunity obtained with MCODE algorithm of Cytoscape were JAK2 and B2M. RT-qPCR and ROC curves confirmed that the hub gene B2M was a specific biomarker of HAPE and had diagnostic value, and single-gene GSEA analysis confirmed that it participated in MHC I molecule-mediated antigen presentation ability decreased, resulting in reduced immunity.

**Conclusion:**

Occurrence and early progression of high altitude pulmonary edema may not be related to inflammation. B2M may be a new clinical potential biomarker for HAPE for early diagnosis and therapeutic evaluation as well as therapeutic targets, and its decrease may be related to reduced immunity due to reduced ability of MCH I to participate in antigen submission.

## 1 INTRODUCTION

High-altitude pulmonary edema (HAPE) is a severe and prevalent illness that affects high-altitude workers and travelers [1]. It affects 0.1%-4% of the population within 1-4 days of reaching high altitude, depending on the altitude [2]. As a result of acute mountain sickness (AMS), severe altitude sickness can develop, which manifests as HAPE [3]. Now most people think that the cause of high-altitude pulmonary edema is that hypoxia causes the contraction of small pulmonary vessels and an increase in pulmonary arterial pressure; the redistribution of circulating blood in the lung increases blood volume and increased vascular permeability [1]. There is still controversy about the pathogenesis of HAPE, and some believe inflammation is also a pathogenic factor [4-6]. Peripheral blood contains immune cells, including lymphocytes, monocytes and neutrophils, which may regulate HAPE. A study has proposed that pulmonary inflammation causes HAPE [7], and hypoxic stimulation causes the increase of tumor necrosis factor (TNF-α), interleukins (IL-1, IL-2, IL-6) and C-reactive protein (CRP) in blood [8, 9]. These inflammatory factors can also be released by immune cells in the blood to invade the lungs and cause pneumonia, which further causes HAPE [10]. However, some studies have reported that individual susceptibility, including clinical susceptibility, sleep and other mental factors, and upper respiratory tract infection and other factors can also lead to HAPE [7, 11, 12]. These are not difficult to verify. Some people who go to high altitudes together have adapted to high-altitude environments, and others have high-altitude pulmonary edema.

Interstitial edema is prevalent in the early stages of acute pulmonary edema, whereas alveolar edema is found in the later stages. Wet rales, wheezing, and dyspnea are not typically found in interstitial pulmonary edema. Therefore, these patients are often misdiagnosed, causing treatment delays. Currently, clinical symptoms and medical images are used to diagnose HAPE routinely. However, it is more challenging to diagnose atypical HAPE. Usually, patients develop atypical HAPE at an altitude of 3000 m, which manifests over a number of days and has distinct symptoms from typical HAPE, such as little to no sputum and non-specific chest x-ray outcomes [13]. As a result, an early and accurate diagnosis of HAPE is more difficult, which may allow the disease to progress in most patients, resulting in limited treatment options and a poor prognosis. Therefore, identifying biomarkers for early HAPE diagnosis is essential for improving patient outcomes.

Noninvasive blood-based biomarkers, which are simple and sensitive tests, may also be critical for personalized diagnosis and treatment of high-altitude pulmonary edema, in which the therapy is matched with the molecular characteristics of the patient. Although previous studies have demonstrated ET-1, soluble kinase domain receptor (sKDR), Corin, angiotensin-converting enzyme, natriuretic peptide (NPs), high-sensitivity troponin T (hs-cTnT), metabolites (C8-neuramide, sphingosine, and glutamine), and sulfotransferase 1A1 (SULT1A1) as diagnostic markers for high altitude pulmonary edema [6, 14, 15]. There are no gold standard diagnostic and therapeutic markers or effective prognostic indicators for patients. Hence, finding novel biomarkers is crucial.

High-throughput technologies have been deployed for the identification of biomarkers [16]. Clinical use of bioinformatics has the potential to improve cancer patient diagnosis, treatment, and prognosis prediction [17]. Additionally, bioinformatics analysis has led to identifying numerous biomarkers for non-cancer disorders [18, 19]. This study aims to identify the core biomarkers of HAPE, understand whether the occurrence and early progression of HAPE are related to inflammatory response, and find new molecular mechanisms for the occurrence and early progression of HAPE.

## 2 MATERIALS AND METHODS

### 2.1 Data Preprocessing and Identification of Differentially Expressed Genes (DEGs)

The Gene Expression Omnibus (GEO) database (https://www.ncbi.nlm.nih.gov/geo) [20] was searched, and two sets of microarray data from the dataset GSE52209 were downloaded for adaptation to the high altitude (n=14) and development of high-altitude pulmonary edema at the same altitude (n=17) within 42-72 hours. The raw data were downloaded as MINiML files.”Limma” package of the R software was utilized to identify DEGs [21]. Volcano plots were then generated using the “ggplot2” package. The top 10 DEGs that were up-regulated and the top 10 DEGs that were down-regulated were shown as heatmaps using the “pheatmap” function of the R package. Genes with p-value < 0.05 and log 2|FC| > 1 were defined as DEGs [22-24].

### 2.2 Functional Annotation and Pathway Enrichment Analysis

“ClusterProfiler” is an R softwore’s package for feature enrichment analysis. GO annotation, KEGG enrichment analysis and Gene Set Enrichment Analysis (GSEA) were carried out using the “clusterProfiler” package. Cellular components, biological processes and molecular functions are all included in the GO functions. The genomic and molecular functions of genes and their correlation with other genes were described using the KEGG pathway analysis. P value < 0.05 was taken as statistically significant [25, 26]. Use the “ggplot2” package to display five GSEA enrichment pathways simultaneously and draw the mountain map to show the enrichment of each pathway. NOM *p*-value < 0.05 and FDR q-value < 0.25 were defined as the criteria for significant results.

### 2.3 Protein-protein Interactions (PPI) Network Analysis of DEGs and Immune Genes for Datasets and Identification of Hub Genes

Protein-protein interaction (PPI) analysis was done by STRING (https://cn.string-db.org/) using a confidence score of > 0.4 to identify PPI pairs [27]. Then, Graphed with R's “igraph”package [28]. The network of proteins was subjected to a cluster analysis using the MCODE plug-in of Cytoscape. The gene cluster with the highest score contained the hub genes [29, 30]. The hub genes of the DEGs and immune genes in the dataset intersect as possible biomarkers and are associated with immune infiltration.

The “Wayne” map with the “Venn-Diagram” package shows the intersection of the immune gene dataset (downloaded from the ImmPort (https://www.immport.org/) database) and DEGs.

### 2.4 Establishment of High Altitude Pulmonary Edema Model

A total of 26 male SD rats weighing (210±20) g were purchased from Daping Hospital, Army Medical University. The standard for feeding was laboratory feeding with a free diet and water, 24°C indoor constant temperature, and a 12-hour light cycle. The Ethics Committee of the Army Medical University approved the experiment.

The experimental animals were kept in the low-pressure chamber (Patent No.: ZL201610421645.1) at a speed of 400 m/min and 12.5 min to the simulated altitude of 5000m. The air pressure in the cabin was -45 - -50 kPa, the humidity in the low-pressure cabin was 40% - 50%, the temperature was kept constant at 24°C, the light cycle was 12 hours, and the food and water were fed freely. The rats were classified randomly into two groups: (1) Control (n = 13): rats not treated with hypoxia or low pressure; (2) HAPE (n = 13): animals were exposed to low pressure and hypoxia for 72 hours.

### 2.5 Determination of Lung Water Content, Weight Change after Modeling, and Lung Coefficient

To determine Lung Water Content (LWC), modeled body weight change, and lung coefficient, the rats were weighed before being subjected to hypobaric hypoxia treatment in two groups (n=6/ group). Precision electronic balance (OHAUS TS-400D; division value, d=1mg; OHAUS, USA) was used for weighing rats. Animals were sacrificed immediately following anesthesia by injecting 2.5% tribromoethanol intraperitoneally. After collecting the lung tissues, the wet weight of the lungs was measured (Quintix223-1CN, division value, d = 1mg; Sartorius, Sartorius Scientific Instruments Co., Ltd., Beijing, China). The tissues were then placed in an oven at a constant temperature of 50° C for 72 hours to obtain their constant weight by Elliot formula [31], LWC percentage water content (%) = [(wet weight - dry weight)/ wet weight] × 100% [32];Modeling weight change = weight after modeling (g) - weight before modeling (g); Lung coefficient = wet weight of both lungs (g)/ body weight (kg) [33].

### 2.6 Hematoxylin-eosin (HE) Staining

The model was constructed as previously mentioned. Following modeling, the lung tissues of three rats from each group were extracted under anesthesia, placed in PBS solution, fixed with 4% paraformaldehyde solution for more than 24 hours, cut, dehydrated, and immersed in wax. These tissues were then embedded, sectioned into 4μm-thick pieces, dewaxed, stained with hematoxylin and eosin, dehydrated, and sealed. After all the processes, a microscopic examination (U-HGLGPS IX71, OLYMPUS, Japan) was carried out, and the data was analyzed.

### 2.7 Immunofluorescence Microscopy Analysis

Immunofluorescence staining was accomplished by deparaffinizing the above paraffin sections, repairing the antigen, and blocking them by drip for 30 minutes. Phosphate buffer solution (PBS) was then diluted the primary antibody before it was incubated at 4°C overnight. Fluorescein-labeled secondary antibodies were then incubated for 50 minutes at room temperature after washing. The sections were incubated for 10 minutes at room temperature in the darkness before the nuclei were counterstained with DAPI. After adding the autofluorescence quench agent for 5 minutes, the slides were then rinsed with running water for 10 minutes. The mounted slides were observed and analyzed using a fluorescence microscope (U-HGLGPS, OLYMPUS, Japan). Aquaporin 5 (AQP5) (NBP2-92926, NOVUS), Inducible nitric oxide synthase (NOS2) (bs-2072R, Bioss), and Endothelin-1 (ET-1) (NB300-526, NOVUS) were labeled by CY3 (Servicebio, GB21303; Servicebio, GB21301) as red fluorescence.

### 2.8 Relative Expression, Correlation Analysis and Verification of Potential Biomarkers

The “PheatMap” of the R software was used to present multi-gene correlation maps, and the “boxplot” function of The “ggplot2” package was used to compare the differentially expressed genes in the dataset.

After modeling the rat (Control: n = 4; HAPE: n = 4), their peripheral blood’s total RNA was isolated for qPCR using the TRIzol kit. Quantus fluorometer was utilized to evaluate the purity of RNA. First, a reverse transcription kit (R323-01, Vazyme) was utilized for the reverse transcription of total RNA. The 2 × ChamQ Universal Master Mix was then added to the pre-amplified cDNA samples (Q711 - 02, Vazyme). The reaction was then carried out on the CFX96 Real-time System (CFX 96 Touch, Bio-Rad). The comparative Ct method was used to determine each sample, and β-actin was a positive control (Table **1**).

### 2.9 Single-gene GSEA Analysis of Hub Genes

GSEA 4.3.2 software was used to perform single-gene GSEA on the hub genes in the immune genes in the dataset [34]. NOM p-value < 0.05 and FDR q-value < 0.25 was considered significant result. The ImmPort database was used to collect 1,793 immune-related genes [35].

### 2.10 Receiver Operating Characteristic (ROC) Curve Analysis of Hub Genes

The “pROC” package was used to analyze the dataset for hub gene ROC curves, and the “ggplot2” package was used to visualize the results [32]. Genes having an AUC > 0.7 were regarded as HAPE-related diagnostic genes.

### 2.11 Drug-gene Interaction

To find drugs that interacted with the validated genes, the Comparative Toxicogenomics Database (CTD, http://ctdbase.org/) was used.

### 2.12 Evaluation of Immune Cell Infiltration and Correlation Analysis of Hub Genes and Infiltrating Immune Cells

The immune cell infiltration matrix was obtained using the gene expression matrix data that was uploaded to CIBERSORT [34], and samples with p < 0.05 were filtered out. Use the “ggplot2” software to generate a histogram of immune cell infiltration percentages and a violin plot to visualize differences in immune cell infiltration. A correlation heatmap of the 22 different types of infiltrating immune cells was made using the “corrplot” package. Spearman correlation analysis was carried out on biomarker and infiltrating immune cells using the “ggstatsplot” package (https://github.com/IndrajeetPatil/ggstatsplot), and the “ggplot2” package was utilized to visualize the results.

### 2.13 Weighted Gene Co-expression Network Analysis (WGCNA)

The “WGCNAR” package was utilized to perform weighted gene co-expression network analysis of the expression profiles of the data sets. To detect any outliers, samples were first clustered. The co-expression network was then obtained using the automatic network construction function. The PickSoft-Threshold was used to calculate the soft thresholding power. Additionally, hierarchical clustering and the dynamic tree-cut function were applied to identify different modules. The Pearson correlation coefficient between the sample vector of variables and the characteristic gene of the module was calculated to determine the strength of the relationship between the clinical characteristics and the modules. We use phenotype and correlation of module characteristic genes to estimate the association between the module and immune cells and identify gene modules associated with specified immune cells.

### 2.14 Statistical Analysis

GraphPad Prism 8.0 software was employed for graphing and statistical analyses. The RT-qPCR data were presented as mean ± standard error of the mean (SEM). The data were analyzed with a two-tailed t-test using Student or Welch t-tests. The difference was statistically significant at *P* < 0.05.

## 3 RESULTS

### 3.1 Data Preprocessing and Identification of DEGs

**Fig. (1):**
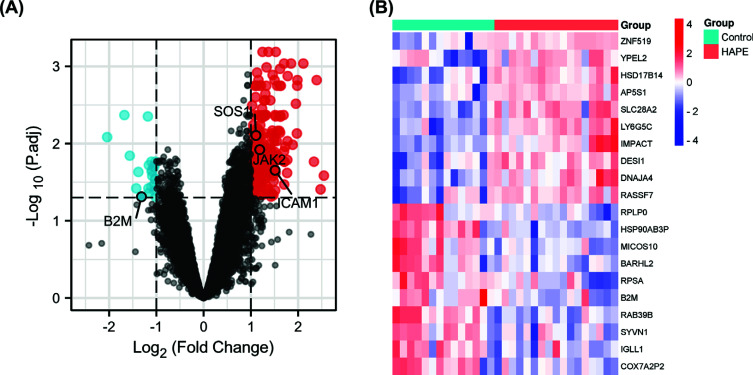
Differentially expressed genes (DEGs) identification in HAPE. (**A**) Volcano visualization of DEGs in the altitude adaptation group and the altitude pulmonary edema group. (**B**) A heatmap visualization of DEGs. The top 10 up-regulated and down-regulated DEGs are represented by rows and samples by columns. DEG expression levels are represented by colors.

The GEO database was searched for a dataset of gene expression profiles (GSE52209) from plateau-adapted plain traveler and HAPE samples. The “Limma” package of the R was used to identify 223 DEGs (Table **2**), there are 200 genes up-regulated and 23 genes down-regulated. “ggplot2” of the “R” was utilized to create a volcanic map to visualize the DEGs (Fig. **1A**), followed by “pheatmap” to create a heatmap of the top 10 up-regulated and down-regulated DEGs (Fig. **1B**).

https://www.ncbi.nlm.nih.gov/pmc/articles/PMC7151213/B17-diagnostics-10-00171

### 3.2 Functional Enrichment Analysis of DEGs

**Fig. (2):**
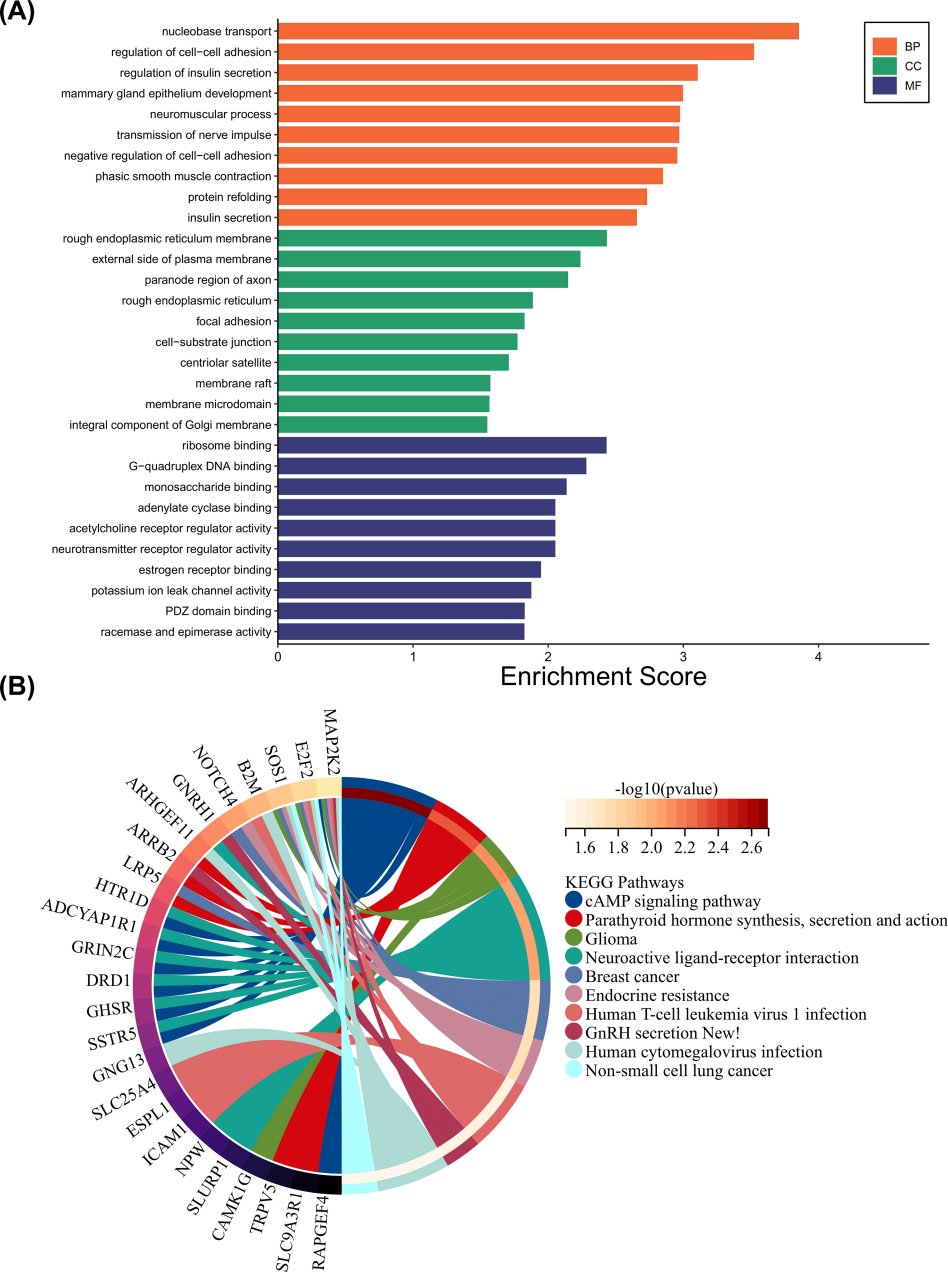
Analysis of DEG enrichment by GO and KEGG pathways. (**A**) Three functional groups were included in the GO analysis: cell components, biological processes, and molecular functions. Each functional group's top 10 pathways were enriched. (**B**) KEGG enrichment analysis of the top 10 enriched pathways.

The DEGs were primarily enriched in nucleobase transport, cell-cell adhesion regulation, and insulin secretion regulation concerning GO biological processes. In the cellular part, the DEGs were mainly found in the rough endoplasmic reticulum membrane, the outer side of the plasma membrane, and the paranode region of the axon. The three most significantly enriched terms in the molecular function group were ribosome binding, G-quadruplex DNA binding, and monosaccharide binding (Fig. **2A**). The KEGG analysis revealed that the DEGs were enriched for a number of pathways, including the cAMP signaling system, the synthesis, secretion, and action of parathyroid hormone, glioma, human T-cell leukemia virus 1, and human cytomegalovirus infection (Fig. **2B**).

**Fig. (3):**
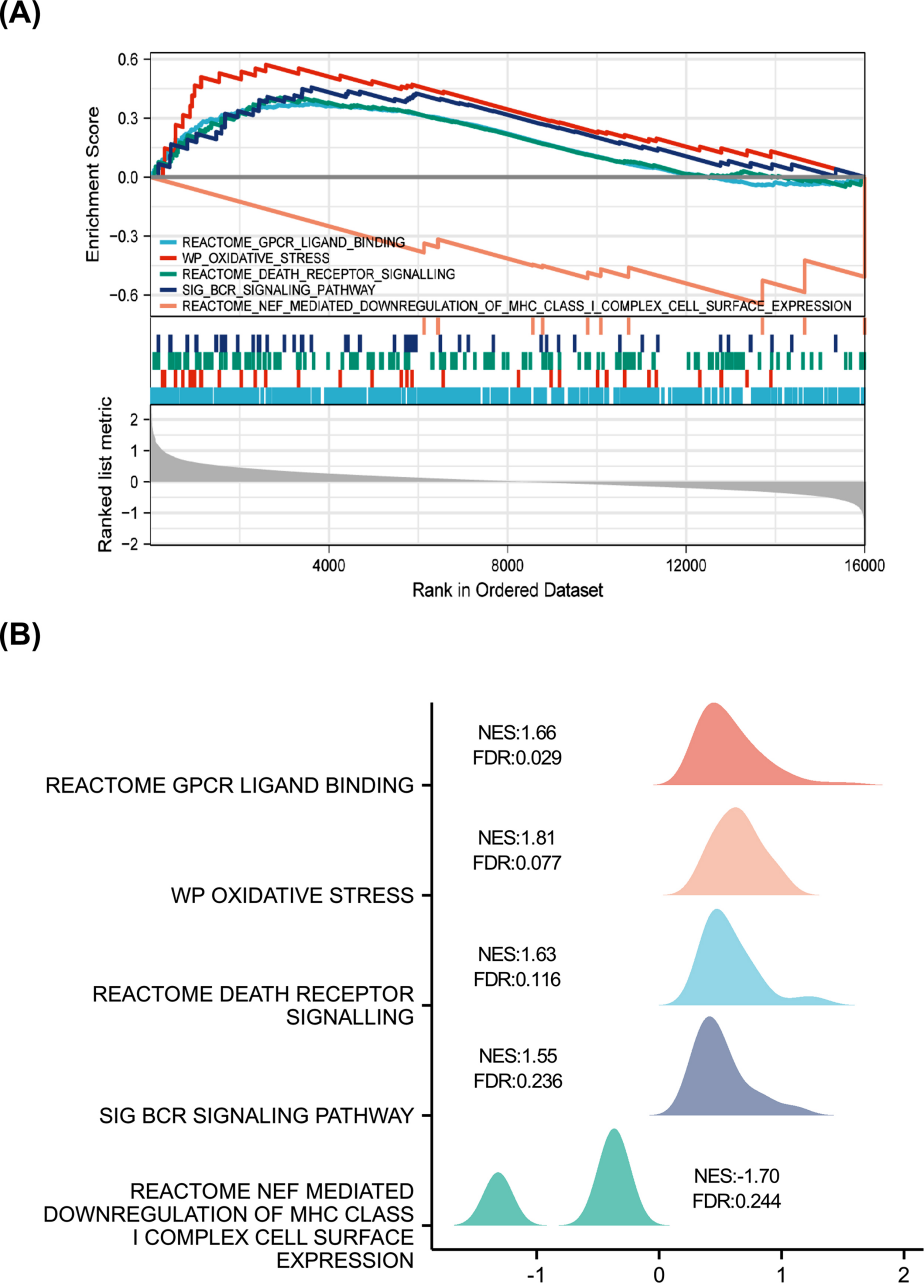
GSEA analysis and GSEA mountain map. (**A**) GSEA analysis of the dataset. (**B**) Map of mountains analyzed by GSEA.

The results of the GSEA enrichment analysis were GPCR ligand binding, oxidative stress, death receptor signaling, BCR signaling pathway, and NEF-mediated downregulation of MHC class I complex cell surface expression (Fig. **3A**). GSEA mountain map showed that NES was positive in the first four pathways, which were up-regulated pathways, and logFC values were also positive in these pathways. In the last pathway, the NES was negative and the pathway was down-regulated, while LogFC, the key component of this pathway, also had a negative value (Fig. **3B**, Supplementary File **1**).

### 3.3 PPI Network Construction and Hub Gene Identification

**Fig. (4):**
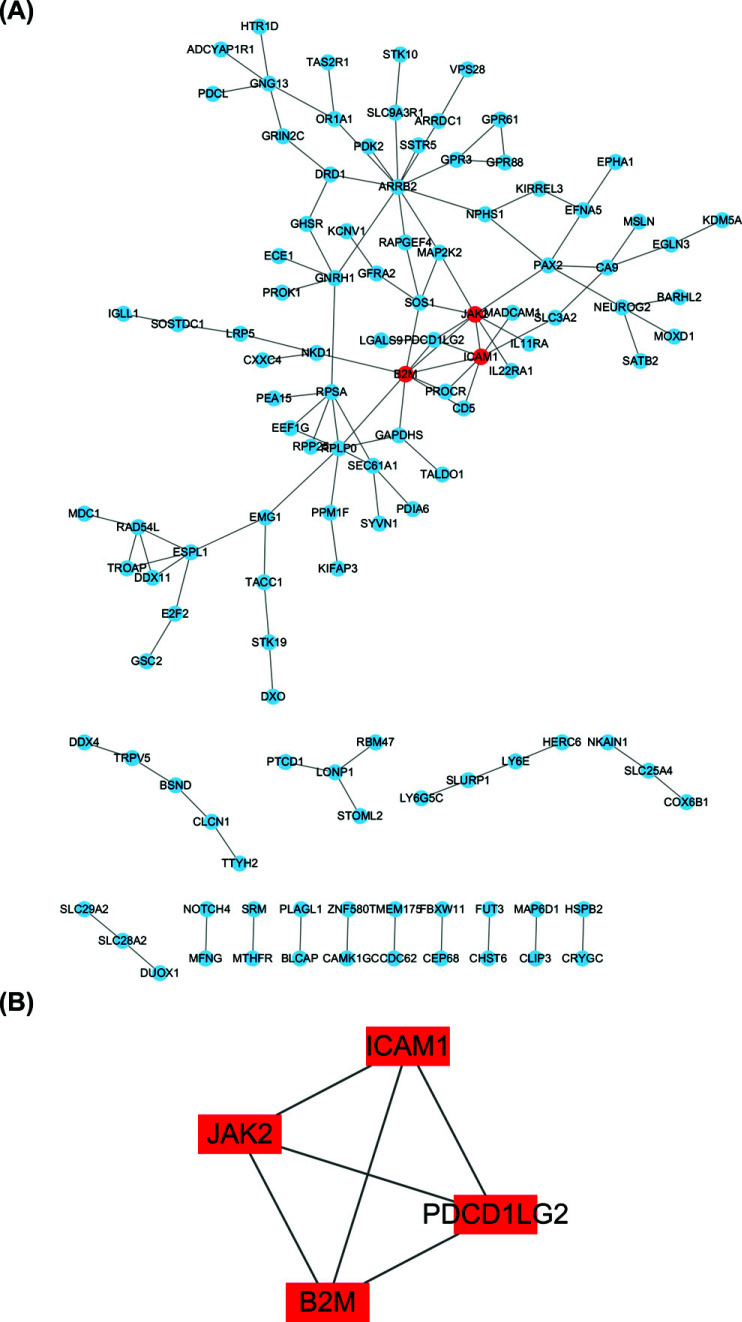
DEG-related PPI network construction, major module analysis, and hub gene identification. (**A**) The whole DEGs PPI network. (**B**) The hub genes and their associated genes that make up the PPI network.

**Fig. (5):**
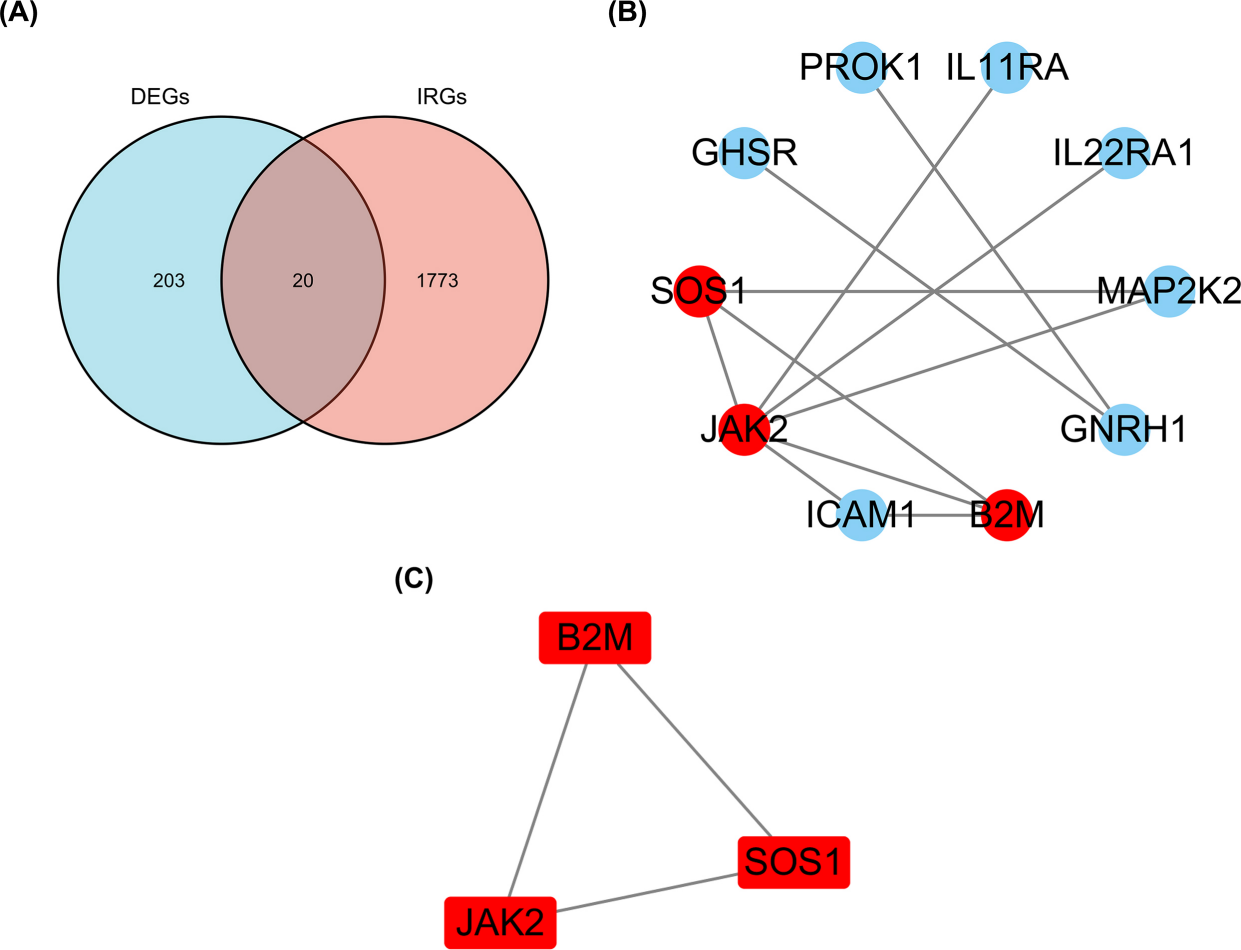
PPI network construction using immune DEGs, module analysis, and hub gene identification. (**A**) Venn diagram of DEGs and IRGs. (**B**) The whole immune DEGs PPI network. **(C)** The hub genes and their associated genes visualized as a PPI network.

As per the node degree scores produced by Cytoscape, B2M, JAK2, ICAM1, and PDCD1LG2 were determined to be the hub genes of the dataset when analyzed and visualized by means of PPI analysis (Fig. **4A**) using Cytoscape on STRING (Fig. **4B**). The PPI network of immune genes in DEGs showed B2M, JAK2 and SOS1 as hub genes (Fig. **5A**-**C**). The intersection of hub genes of DEGs and hub genes of immune genes yielded B2M and JAK2 as hub genes.

### 3.4 Construction of *in vivo* Models

**Fig. (6):**
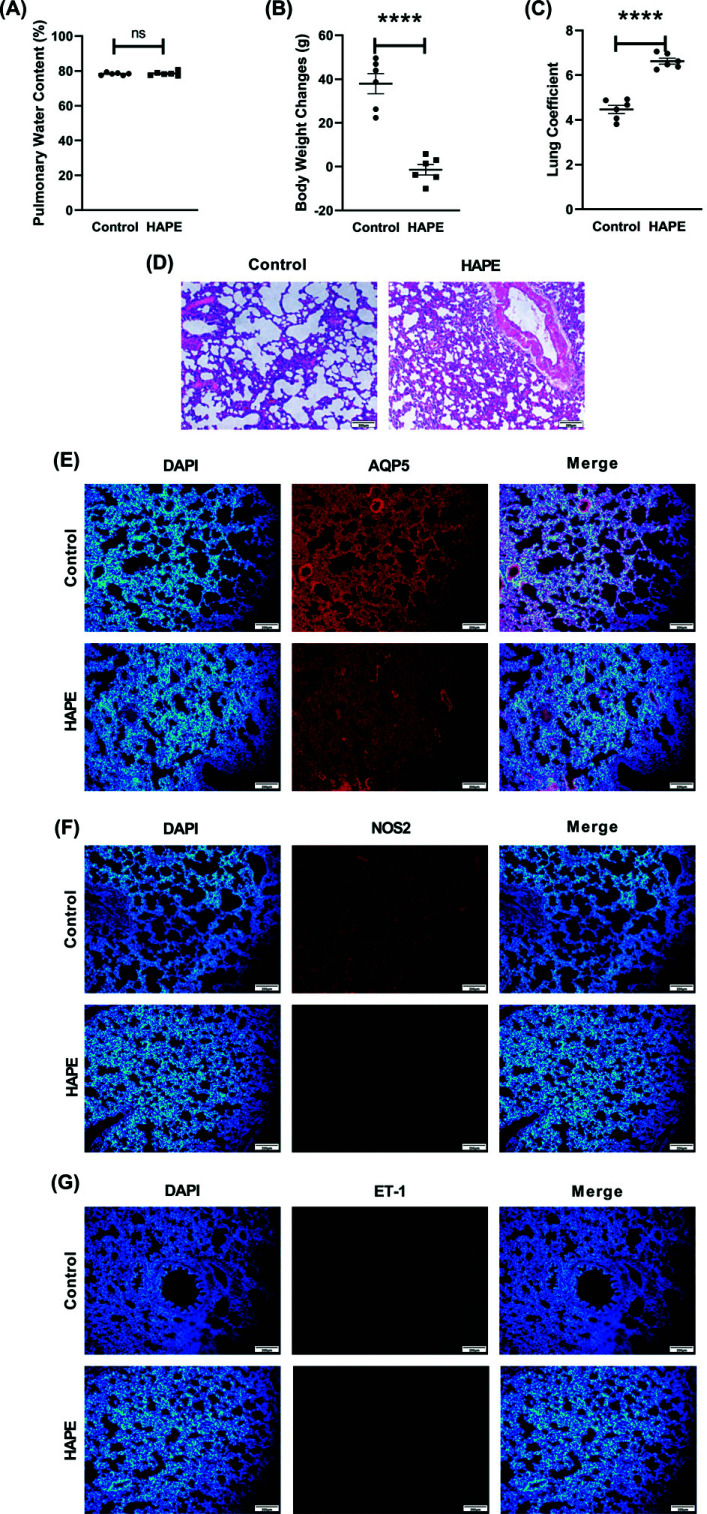
**Lung water content, weight change after modeling, and determination of lung coefficient. (A)** The changes in body weight (**B**) Pulmonary water content and **(C)** lung weight coefficients of rats in the control group and model group were compared (n=6). **(D)** The figure shows HE staining (n=3), Scale bar, 200μm. **(E-G)** Lung tissue immunofluorescence images that are representative of the group were stained for AQP5, INOS, and ET-1 in red and DAPI in blue (n = 3). The data are presented as mean ± SEM. For the comparison between the two groups, *p < 0.05 and **p < 0.01 were used.

In this study, the lung water content did not change significantly. According to previously reported studies, acute hypoxic exposure can result in acute diuretic, cause dehydration, and result in weight loss, while pulmonary edema may not be obvious. Plateau low oxygen exposure can disrupt the body's fluid balance. Alpine hypoxia cases with higher ventilation can cause significant moisture loss resulting in reducing the water content, thus altering the water metabolism [36, 37]. This study supports the findings of the previously reported studies. Calculations of the lung coefficient revealed a considerable variation in edema (Fig. **6A**-**C**).

HE staining revealed that the alveolar cavity of rats in the pulmonary edema group contained an abundance of exudate compared to rats in the control group, alveolar wall capillaries were congested, and the alveolar septum was thickened (Fig. **6D**).

Decrease in AQP5 results in high-altitude pulmonary edema [38]. This study demonstrated that in the modeling group, AQP5 fluorescence was decreased, expression was downregulated, AQP5 channels were closed, and pulmonary edema was developed in contrast with the control group. iNOS, also known as NOS2, catalyzes the production of NO, and a decrease in iNOS results in a reduction in NO content, which in turn leads to pulmonary hypertension and increased pulmonary vascular permeability, exceeding the compensatory capacity of the body, which ultimately leads to HAPE [39]. The iNOS enzyme in lung macrophages and neutrophils increases vascular permeability; this finding was confirmed in studies in iNOS knockout mice [40]. Increased microvascular permeability of ET-1 causes pulmonary edema [41]. According to the findings of this study, ET-1 fluorescence did not increase significantly, and these results may be biased due to the amount of time the lung tissues are exposed to air and the experimental error (Fig. **6E**-**G**).

### 3.5 Hub Gene Expression Comparison and Gene Correlation

**Fig. (7):**
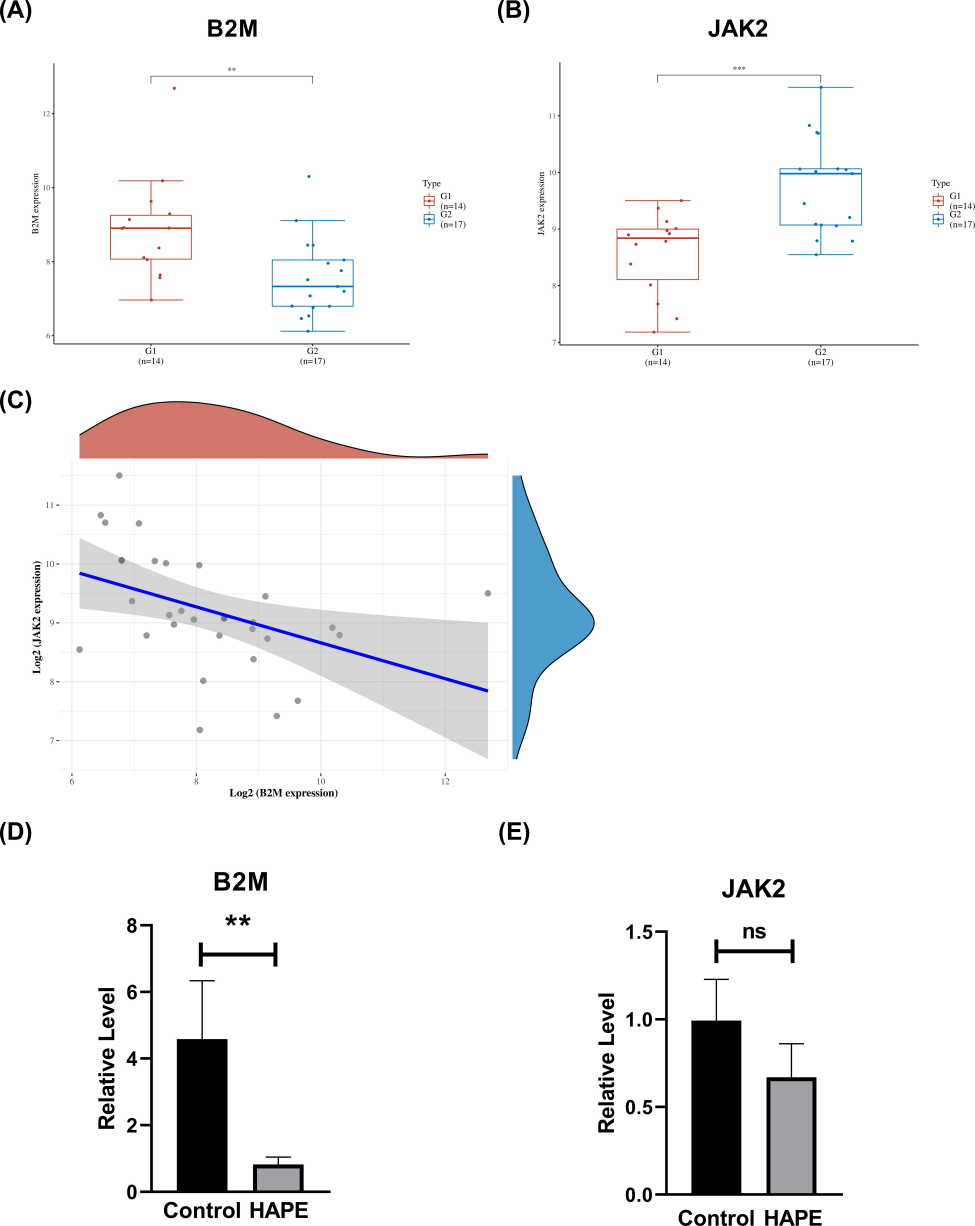
Hub gene expression comparison and gene correlation. (**A**) Hub gene expression distribution in different groups. T-test was passed on both groups of samples. G1: Control group, G2: HAPE group. (**B**) Correlation plot of two genes. (**C**) Hub gene validation using RT-PCR between the Control and HAPE groups. (**D** and **E**) Data are presented as mean ± SEM. Each experiment was repeated in triplicate. The asterisk (*P) denotes the significance level (*p<0.05, **p<0.01).

Compared with the high altitude adaptation group, the HAPE group JAK2 mRNA expression was up-regulated and the expression of B2M was down-regulated (Fig. **7A** and **7B**). The JAK2 and B2M expression levels were significantly inversely correlated with each other (Fig. **7C**). The JAK2/STAT3 pathway is a pro-inflammatory pathway [42], and B2M is also positively correlated with the body's immune function [43], so the results suggest that the occurrence and early progression of HAPE may not be related to inflammation.

### 3.6 Potential Biomarker Expression by RT-qPCR

JAK2 and B2M were regarded as hub genes. RT-qPCR was performed to validate the selected biomarkers in samples after their selection. Rats with high-altitude pulmonary edema had significantly lower levels of B2M expression compared to control rats, which showed consistency with the bioinformatics analysis and could be considered as a biomarker of high-altitude pulmonary edema (Fig. **7D** and **7E**). B2M protein is the component protein of antigen presentation MCHI, MCHI is an important protein involved in antigen presentation, so the decrease in B2M expression will lead to a decrease in antigen presentation, and the patient's immunity will be reduced. This is consistent with the previous GO, KEGG, and GSEA analyses.

### 3.7 Single-gene GSEA Analysis of the Hub Gene

**Fig. (8):**
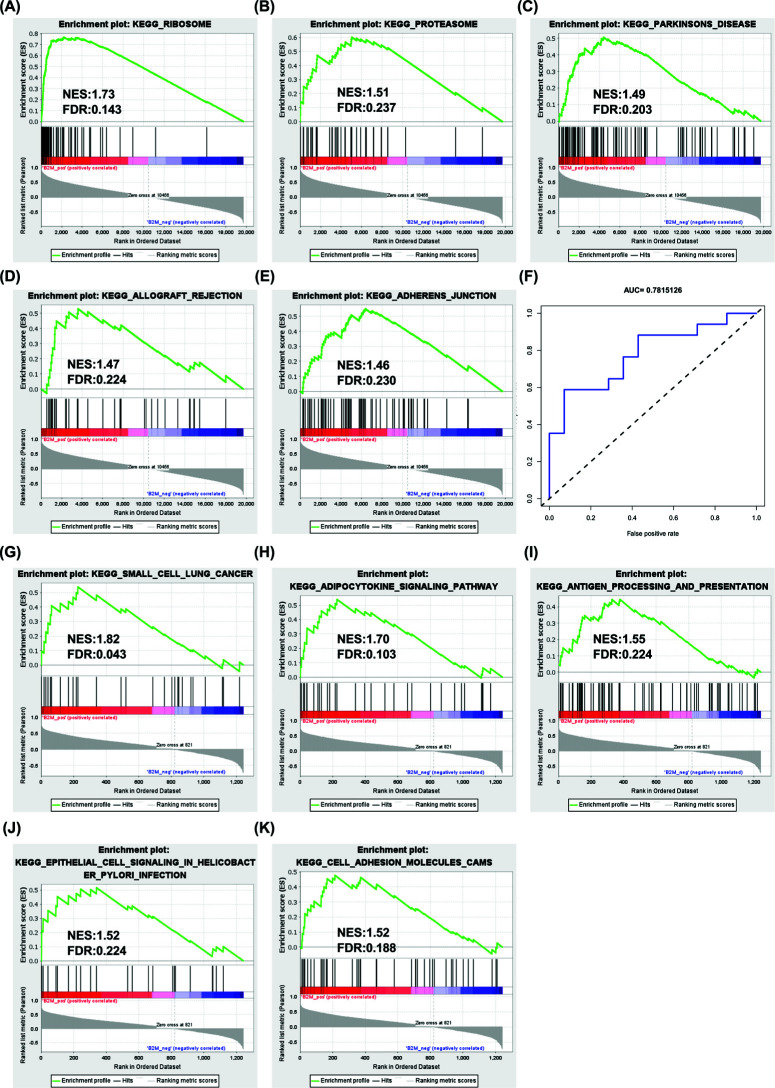
ROC curve analysis and Single gene GSEA analysis of the hub gene. **(A-E)** Single gene GSEA analysis of B2M in the dataset. **(F)** Analysis of ROC curves of the hub gene. AUC: Area under the curve. **(G-K)** Single gene GSEA analysis of B2M in the immune gene dataset.

The enrichment results of the hub gene single gene GSEA of the dataset were synthesis of the proteasome and ribosome, parkinson disease, allograft rejection, and adherens junction pathways (Fig. **8A**-**E**). The enrichment results of the Single-gene GSEA of the immune genes were small cell lung cancer, adipocytokine signaling pathway, antigen processing and presentation, epithelial cell signaling in helicobacter pylori infection and cell adhesion molecules CAMs pathways (Fig. **8G**-**K**). The enrichment results revealed that the hub gene B2M was involved in immune regulation and positively correlated with antigen presentation. The results showed that compared with the plateau hypoxia adaptation group, the antigen presentation ability of the high-altitude pulmonary edema group may be reduced, and its immunity may be reduced.

### 3.8 ROC Curve Analysis of Hub Genes

In order to assess the diagnostic utility of hub genes, the ROC curve was drawn, and the area under the curve (AUC) was calculated. The AUC of the hub gene was 0.782 (Fig. **8F**). Therefore, the hub genes have a higher diagnostic value as a biomarker. B2M can be used as a serum screening biomarker in the altitude adaptation group and the early HAPE group.

### 3.9 Drug-gene Interaction Analysis

A total of 192 drugs acting on B2M were identified using the CTD database (Supplementary File **2**). The results show that there are multiple drugs that affect the expression of mRNA and protein of B2M, and B2M can be targeted for treatment.

### 3.10 Evaluation of Immune Cell Infiltration and Correlation Analysis of Hub Genes and Infiltrating Immune Cells

**Fig. (9):**
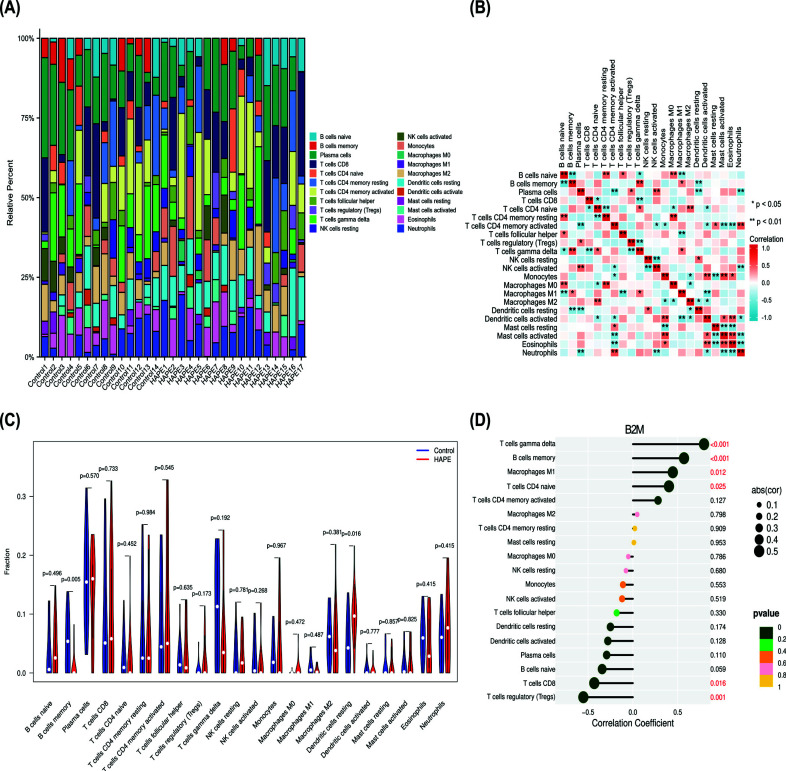
Assessment of immune cell infiltration and correlation analysis, as well as the infiltration immune cell correlation of Hub genes. (**A**) Bar chart of infiltration percentage of 22 immune cells; (**B**) heat map of 22 kinds of immune cell invasion; (**C**) Violin plot of 22 immune cell infiltration compared with control group; (**D**) Correlation map of single gene immune cell invasion.

The bar chart shows the percentage of 22 immune cell infiltration (Fig. **9A**). The results of the bar plot and violin plot together demonstrate that the level of dendritic cells resting was substantially higher as compared to the control group, and the level of B cell memory was lower as compared to the control group (Fig. **9C**). Immune cell correlation plots for single genes show the correlation of 22 immune cells in the form of heatmap (Fig. **9B**). Investigating the relationship between hub genes and immune cells it was revealed that B2M and immune cells such as T cells gamma-delta, B cells memory, Macrophages M1, T cells CD4 naive, and CD4 memory activated T cells were positively correlated. It was negatively associated with the regulatory T cells (Tregs) and T cells CD8 (Fig. **9D**). B2M expression was positively correlated with memory B cell levels and negatively correlated with resting dendritic cell levels, which was consistent with previous results, indicating that relative to the altitude adaptation group, the level of immune effector cells decreased in the HAPE group, the expression of B2M decreased, and immunity decreased.

### 3.11 Construction of Weighted Co-expression Network and Key Modules Identification

**Fig. (10):**
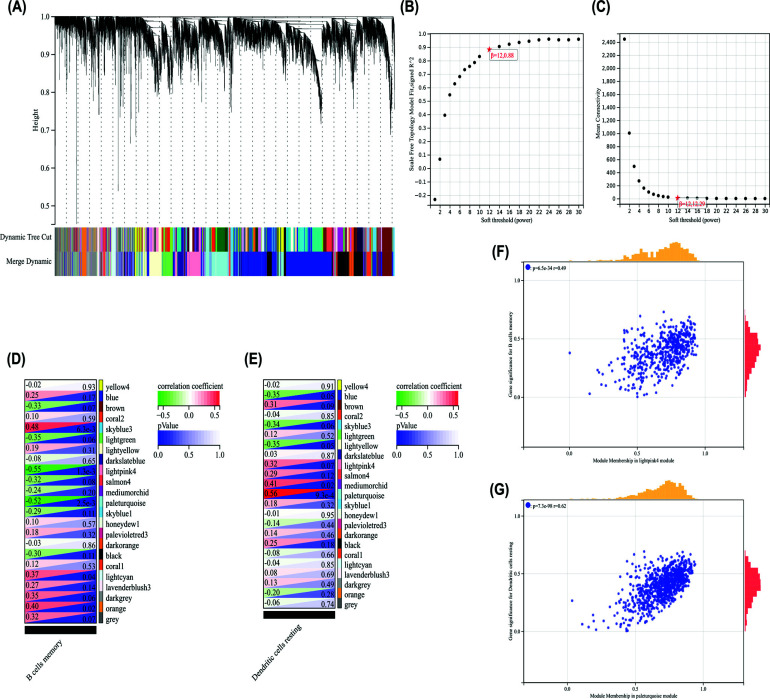
B cell memory and DC resting related gene signature identification. (**A**) Genes are grouped in a dendrogram based on topological overlaps. Modules that corresponded to different colors were shown. (**B** and **C**) Network topology analysis using the scale-free fit index (left) and mean connectivity (right) for different soft-thresholding powers. (**D** and **E**) A total of 23 modules (nongrey) were identified. The lightpink4 module (r = -0.55, *P* = 1.3e^-3^) and the pale turquoise module (r =0.56, P = 9.3e^-4^) had the highest correlation and were considered the most correlated with memory B cells and resting DC cells. (**F** and **G**) The relationship between the significance of a gene and its membership in the most relevant memory B cell and resting DC cell module.

To cluster the samples, Pearson’s correlation coefficient was used and no outlier was detected (Fig. **10A**). The soft threshold was set to 12 to construct a scale-free network (Fig. **10B** and **C**). On the basis of average hierarchical clustering and dynamic tree clipping, 23 modules were then identified. The paleturquoise module was most associated with resting dendritic cells, while the lightpink4 module was most associated with memory B cells. Consequently, the lightpink4 module with 538 genes and the pale turquoise module with 896 genes were subjected to further analyses (Fig. **10D** and **E**). The gene significance and module membership of the genes in the lightpink4 module in memory B cells and in the paleturquoise module in resting dendritic cells exhibited a high correlation (Fig. **10F** and **G**). This result indicates that resting dendritic cells and memory B cells have the function and phenotype of the paleturquoise module genes and the lightpink4 module genes, respectively.

### 3.12 GO and KEGG Analysis of Immune Cell-related Module Genes

**Fig. (11):**
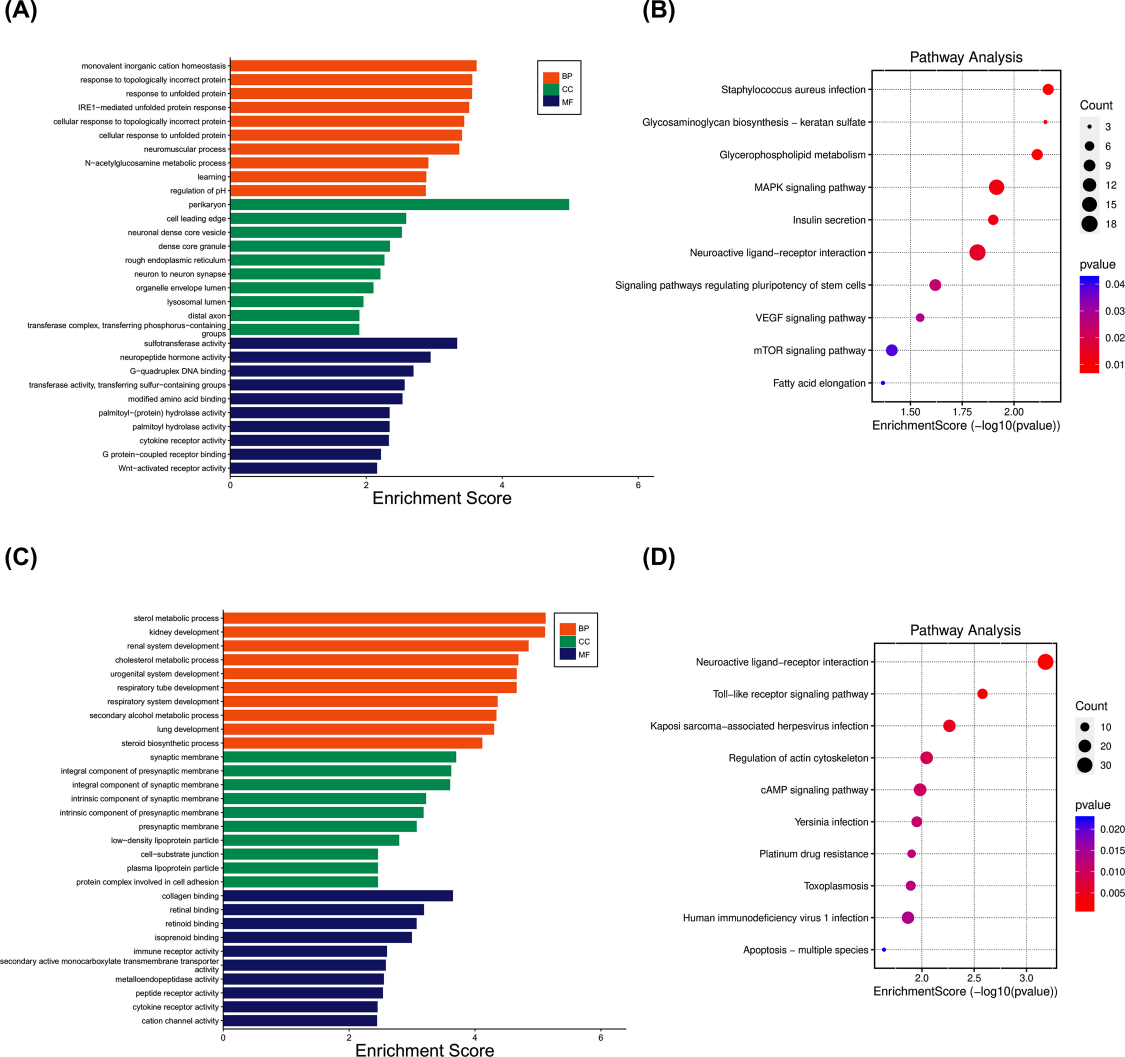
GO and KEGG enrichment analyses of the most relevant module genes for memory B cells and resting dendritic cells. (**A** and **B**) GO and KEGG analyses of the memory B cell module genes that are most relevant (**C** and **D**) GO and KEGG analyses of the most relevant module genes of resting dendritic cells.

The genes in the lightpink4 and pale turquoise modules underwent GO and KEGG analyses and the results are shown in the figure. These genes are primarily involved in inflammatory, infectious, and cell death pathways (Fig. **11**). The results showed that the main functions of memory B cells and dendritic cells were related to inflammation, infection and cell death relative to the control group, and combined with previous studies at the level of memory B cells and dendritic cells, it was indicated that the level of inflammation in patients with HAPE was reduced relative to the altitude adaptation group.

## 4 DISCUSSION

High altitude pulmonary edema (HAPE) is a severe high-altitude disease with a certain fatality rate, and some cases have a poor prognosis after the onset. Early HAPE is interstitial pulmonary edema, the symptoms are not obvious, not specific, it is difficult to diagnose by traditional methods, patients may miss the best diagnosis and treatment time and the prognosis is not good, causing sequelae, so a method that can be diagnosed quickly at an early stage is particularly important. Its treatment is mainly symptomatic treatment, and there are few specific diagnostic and treatment markers in the clinic. At present, its pathogenesis is still controversial, most studies believe that inflammation is not its pathogenesis, but some studies still believe that inflammation can lead to HAPE.

In view of the difficulty of early diagnosis of HAPE, the single treatment method, and the lack of diagnosis and treatment biomarkers, we analyzed the biomarker mRNA detected in peripheral blood by bioinformatics method, and used this method to explore whether the pathogenesis of HAPE is related to inflammation for the first time. Peripheral blood detection mRNA has high sensitivity and low trauma, which is suitable for rapid early diagnosis of HAPE, indicating the progress and prognosis of HAPE treatment.

Download the GSE52209 dataset in the GEO database, which contains two sets of data, the altitude adaptation group and the high-altitude pulmonary edema group, and use these two sets of data for bioinformatics analysis. Then GO, KEGG, and GSEA analyses were done to determine which functions and signaling pathways of HAPE were enriched compared with the high-altitude adapt group. KEGG functional data analysis showed that these DEGs were related to metabolic and infection pathways (Fig. **2B**), and further GSEA analysis showed that the dataset may be related to metabolism, oxidative stress, death receptor signaling pathway, BCR signaling pathway, and downregulation of antigen presentation pathway involving MCH I molecules (Fig. **3**). These studies showed that compared with the control group, the metabolism, oxidative stress, and apoptosis of the HAPE group were enhanced, the antigen presentation ability involved in MCH I molecules decreased, and there may be activation of infection pathways. Studies have shown that HAPE is associated with increased oxidative stress, metabolism and apoptosis, and is also prone to secondary infection, which is consistent with our findings [44, 45].

Through Cytoscape's “MCODE” algorithm, we analyzed the immune gene of the dataset and the hub gene of DEGs, and then obtained biomarkers related to immune infiltration after intersection (Figs. **4** and **5**). The rat model of HAPE was established, and then the biomarkers of HAPE were verified by QPCR (Fig. **6**). The obtained biomarkers related to immune infiltration were analyzed for ROC curves and single-gene GSEA (Fig. **8**), and then drugs acting on it were searched. Through the above analysis, we assessed that B2M has a good diagnostic value, and the single-gene GSEA results of the dataset and the immune genes in it show that B2M is positively correlated with immunity and antigen presentation. The results are consistent with previous GO, KEGG, and GSEA analyses. Compared with the control group, the above analysis results showed that the expression of immune invasion biomarker B2M in patients with HAPE decreased and there was no obvious inflammatory response, and their antigen presentation ability was reduced, and their immunity may be reduced.

Finally, we used “CIBERSORT” algorithm to analyze immune infiltration and the correlation between significant immune invasive cells and B2M (Fig. **9**), and then used “WGCNA” analysis to obtain module genes related to significant immune invasive cells and did enrichment analysis (Fig. **10**). The results showed that compared with the control group, the HAPE group had decreased memory B cells, resting dendritic cell increase, and B2M was proportional to the level of memory B cells, indicating reduced immunity, which was consistent with the previous conclusion. The enrichment analysis results of the module genes most associated with memory B cells and resting dendritic cells were associated with inflammation, infection, and cell death (Fig. **11**), and were also consistent with the previous GO, KEGG, and GSEA enrichment assays.

β2 microglobulin (B2M) is a component of the MHC I, which plays a key role in the immune response to antigens [46, 47]. Cell surface receptors on CD8^+^ T and NK cells activate immune responses when they recognize it [48]. A reduction in this protein may hinder NK cells and CD8^+^ T cells in their ability to present antigens. According to the study, B2M gene disruption results in the loss of MHC class I surface expression [49]. It was proved by this study that B2M is necessary for the formation of MCH I. Studies have shown that B2M can cause immune rejection in the body, and knockout of B2M can reduce immune rejection [50]. Studies have shown that B2M expression levels do not appear to be affected by hypoxia [51], as hypoxia is not the cause of B2M degradation and may be used as a specific biomarker for early screening, treatment and prognosis of highland pulmonary edema in plateau areas.

When re-infection occurs, memory B cells, a subtype of B cells that develops within the germinal center after primary infection, are crucial for producing faster and more potent antibody-mediated immune responses. The present study found a decrease in the level of memory B cells, which may be related to the regulation of MAPK signaling pathway A decrease in memory B cells has been reported to be associated with autoimmune diseases [51, 52]. Costimulatory molecules, such as CD86, are needed by dendritic cells (DCs) for successfully activating T cells and eliciting adaptive immunity as dendritic cells have an antigen-presenting role When DCs are at rest, they express very little CD86, but when they are stimulated with LPS, their expression levels are increased. Ubiquitination is a key mechanism of DCs to control CD86 expression and regulate its Ag presentation function. Resting Dendritic cells proliferate, weaken the immune system, and easily induce infection [53]. This is consistent with the results we got. There was no significant change in B2M *versus* plain areas among people who adapted to the plateau. Studies have shown impaired peripheral T cell activation in travelers at high altitudes, with no significant change in the number of B cells [54]. Combined with our findings, this may indicate a further reduction in T cell activation and a further decrease in effector B cell levels in patients with HAPE relative to those in the plateau adaptation group.

It has been reported that susceptibility to HAPE is related to EGLN1 gene polymorphisms, which can lead to abnormally elevated HIF-1α in a normoxic state. HIF-1α is higher in hypoxia, which can cause high-altitude pulmonary edema, and HIF-1α may cause inflammation [55]. However, our study did not conclude that excessive inflammation is involved in the development of HAPE because gene polymorphisms result from different genetic susceptibility of individuals. The probability of this inflammation caused by individual susceptibility is very low. There are many studies that show that individual susceptibility plays an important role in the onset of HAPE, including genetic susceptibility, spirit, sleep and other factors, which will lead to different probabilities of HAPE [7, 11, 12].

Targeted therapy is currently a popular treatment method, which has good efficacy and quick effect. Studies have shown that syngeneic mice with genetically deleted B2M develop resistance to PD-1 immunotherapy [56]. Gene therapy for B2M deficiency is an important therapeutic and medication option for the treatment of malignant tumors [56, 57]. This study may provide a rationale for drug research targeting B2M. The CTD database search depicted 192 drugs that may interact with B2M, and this study provides a theoretical basis for the research of drugs targeting B2M (Supplementary File 2). So as to achieve a better effect of treating high altitude pulmonary edema.

From this, we conclude that HAPE patients are more susceptible to co-infection due to decreased memory B cell levels, resting dendritic cell increase, impaired antigen presentation ability of MCHI. Participation, decreased immune function, and HAPE biomarkers are B2M and decreased in the peripheral blood of HAPE patients. However, it also has some limitations. First, we only detected 2 hub genes, and one of them was verified by RT-qPCR. Then, the molecular pathways implicated in the pathogenesis of HAPE were not comprehensively reviewed. To enhance the early diagnosis, treatment, and prognosis improvement of HAPE patients, the hub genes, associated molecules, and pathways may be of major significance in the onset, progression, and patient prognosis of HAPE. However, clinical experimental research is still required to confirm these results further.

## CONCLUSION

Our study found that the occurrence of HAPE may not be related to inflammation, B2M may be a specific biomarker of HAPE, and its decrease may be related to reduced immunity due to the reduced ability of MCHI to participate in antigen submission. Our research may provide a new theoretical basis for the pathogenesis of HAPE, B2M may be a biomarker for the early diagnosis, treatment and prognosis of HAPE and used in clinical practice, and B2M may also be a targeted therapeutic molecule for HAPE.

## Figures and Tables

**Table 1:** Amplification of quantitative real-time PCR data using specific primers.

**Gene**	**Forward (5'-3')**	**Reverse (5'-3')**
B2M	GCTCACACTGAATTCACACCC	CATGTCTCGGTCCCAGGTG
Jak2	TCCACCCAATCATGTCTTCCAC	AAATCATGCCGCCACTGAGC
β-actin	AACCTTCTTGCAGCTCCTCC	TACCCACCATCACACCCTGG

**Table 2:** Identification of DEGs in High altitude pulmonary edema patients’ integrated microarray data.

**DEGs**	**Gene Terms**
Up-regulated	EGLN3 NAXE CA9 TTLL4 FBXW11 SLC28A2 CAMK1G MOXD1 TACC1 EFCAB3 DXO PEX10 PDCL HSD17B14 LY6G5C KIRREL3 PDIA6 GZMM PGBD5 PCYOX1L ECE1 SEC61A1 IMPACT BSND IL11RA TM9SF1 BLCAP KIFAP3 IMPDH1 DPCD DNAJA4 CLCN1 GUCD1 NOTCH4 TMEM161A PDK2 DEFB118 CECR7 DDX11 CRYGC HSH2D MTCL1 SSTR5 PTCD1 MBL2 ANKH CHST6 MTHFR ARRDC1 SLC39A5 PIP4P1 CCDC62 GHSR SLC3A2 LRP5 PAX2 HAX1 TROAP NRBF2 LOC388242 TTC7A DESI1 OPA3 HOXD4 HSPB2 SOS1 ARRB2 SPNS1 OR1A1 KIAA2013 CAP1 SATB2 SLC9A3R1 DRD1 TMEM175 KDM5A RASSF7 CLIP3 PAGR1 SOX7 SALL2 NKAIN1 EIPR1 VPS28 STK10 ASCL3 UBOX5 JAK2 MAP2K2 WWC2 SCAMP3 ACKR1 LY6E ARMC9 KCNV1 GRIN2C PDCD1LG2 GATAD2B FOXB1 NKD1 LINC01006 MDC1 HERC6 LONP1 GCKR MFNG KCNK9 UNC50 CASKIN1 CD5 SLURP1 RPP25 KRT35 KRT86 IL22RA1 PPM1F EVA1B ADCYAP1R1 E2F2 PTPN18 GNRH1 CNTNAP1 ZC3H12A POM121L8P AP5S1 RAD54L GORASP2 UBA52P3 DBNDD1 MVK WBSCR23 TRPV5 EFNA5 SLC29A2 ARHGEF11 GPR61 ODF3L1 SRM RAPGEF4 RBM47 ICAM1 NMRK1 GPR3 PROK1 ZNF703 HTR1D ZNF496 STK19 LYPLA2 CXXC4 EMG1 PROCR PEA15 TTYH2 ESPL1 MADCAM1 ZNF519 GPR88 MAP6D1 TNNI1 NPHS1 YJEFN3 APOL5 NRIP2 LOC391813 PNMT EPHA1 DDX4 SLC25A4 GNG13 LMF1 GFRA2 GSC2 ROGDI CALCOCO2 CYREN DUOX1 HCG4 STOML2 FUT3 PLAGL1 RNF167 MLF2 RAB9B GAPDHS ZNF580 YPEL2 CEP68 LGALS9 MT1F TAS2R1 MSLN TALDO1 NCKAP1L NEUROG2 TSPAN10 BPIFB2 LGALS7 IGFLR1 TMEM196
Down-regulated	HSP90AB3P RAB39B RPLP0 MICOS10 EEF1G NCMAP COX7A2P2 SOSTDC1 SNHG12 IGKC FAM171A2 RPSA YIF1A HECTD2 SIGLEC17P NIPA1 AQP12A BARHL2 SYVN1 NPW IGLL1 COX6B1 B2M

## Data Availability

The study was based on gene expression profile data (GSE52209) from the Gene Expression Omnibus (GEO) database. The datasets used and/or analyzed during the current study are available from the corresponding author upon reasonable request.

## LIST OF ABBREVIATIONS

HAPE: High Altitude Pulmonary Edema
GEO: Gene Expression Omnibus
DEGs: Differentially Expressed Genes
GO: Gene Ontology
KEGG: Kyoto Encyclopedia of Genes and Genomes
GSEA: Gene Set Enrichment Analysis
qRT-PCR: Real-time Fluorescence Quantitative PCR
AMS: Acute Mountain Sickness
sKDR: Soluble Kinase Domain Receptor
NPs: Natriuretic Peptide
hs-cTnT: High-sensitivity Troponin T
SULT1A1: Sulfotransfersae 1A1
PPI: https://string-db.org/Protein-protein Interaction
LWC: Lung Water Content
PBS: Phosphate Buffer Solution
AQP5: Aquaporin 5
NOS2: Inducible Nitric Oxide Synthase
ET-1: Endothelin-1
HE: Hematoxylin–eosin
PBS: Phosphate Buffer Solution
SEM: Standard Error of Mean
B2M: β2 Microglobulin
MHCI: Major Histocompatibility Complex Class I
JAK2: Janus Kinase 2
AUC: Area Under the Curve
DCs: Dendritic Cells
CTD: Comparative Toxicogenomics Database
WGCNA: Weighted Gene Co-expression Network Analysis

## SUPPLEMENTARY MATERIAL

In addition to the published article, supplementary material is available on the publisher's website.
